# Iterative screen optimization maximizes the efficiency of macromolecular crystallization

**DOI:** 10.1107/S2053230X18017338

**Published:** 2019-01-24

**Authors:** Harrison G. Jones, Daniel Wrapp, Morgan S. A. Gilman, Michael B. Battles, Nianshuang Wang, Sofia Sacerdote, Gwo-Yu Chuang, Peter D. Kwong, Jason S. McLellan

**Affiliations:** a Department of Biochemistry and Cell Biology, Geisel School of Medicine at Dartmouth, Hanover, NH 03755, USA; b Vaccine Research Center, National Institute of Allergy and Infectious Diseases, National Institutes of Health, Bethesda, MD 20892, USA

**Keywords:** automated liquid handling, crystallization screening, macromolecular crystallography, Sweet16

## Abstract

Advances in X-ray crystallography have streamlined the process of determining high-resolution three-dimensional macromolecular structures. However, a rate-limiting step in this process continues to be the generation of crystals that are of sufficient size and quality for subsequent diffraction experiments. Here, iterative screen optimization (ISO), a highly automated process in which the precipitant concentrations of each condition in a crystallization screen are modified based on the results of a prior crystallization experiment, is described. After designing a novel high-throughput crystallization screen to take full advantage of this method, the value of ISO is demonstrated by using it to successfully crystallize a panel of six diverse proteins. The results suggest that ISO is an effective method to obtain macromolecular crystals, particularly for proteins that crystallize under a narrow range of precipitant concentrations.

## Abstract 2

An automated, high-throughput crystallographic screening method is presented that has successfully been used to crystallize a panel of six diverse proteins. Iterative screening optimization is highly customizable and easy to implement for the efficient and thorough navigation of chemical space.

## 1. Introduction   

Macromolecular X-ray crystallography has undergone dramatic advances since the crystal structure of myoglobin was first reported (Kendrew *et al.*, 1958 ▸). High-throughput microbatch screening, in conjunction with automated liquid-handling and imaging devices, now allows relatively small volumes of a protein sample to be evaluated for crystallization in hundreds or thousands of crystallization conditions simultaneously (Chayen *et al.*, 1990 ▸, 1992 ▸). Once favorable crystallization conditions have been identified, diffraction experiments can be performed; complete data sets can often be collected in a matter of minutes owing to the use of high-flux synchrotron beams and single-photon-counting detectors (Hendrickson, 2000 ▸; Casanas *et al.*, 2016 ▸). Diffraction data can then be rapidly processed and structures determined with the use of software packages such as *CCP*4, *PHENIX* and *Coot* (Winn *et al.*, 2011 ▸; Adams *et al.*, 2002 ▸; Emsley *et al.*, 2010 ▸). Thanks to these technological advances, it has become possible for crystallographers to generate high-resolution molecular models in days or weeks under ideal conditions.

Despite these improvements, a rate-limiting step in most crystallographic experiments continues to be the determination of chemical conditions that facilitate crystal nucleation and growth (Smyth & Martin, 2000 ▸; McPherson & Cudney, 2014 ▸). A number of strategies have been proposed and implemented to expedite this process, with varying degrees of success. For example, sparse-matrix screening and free-interface diffusion are two techniques that have been used to explore large swaths of chemical space while still requiring minimal quantities of reagents (Salemme, 1972 ▸; Jancarik & Kim, 1991 ▸; Cudney *et al.*, 1994 ▸). Both of these methods have been heavily utilized for crystallographic screening, but each suffers from disadvantages. Sparse-matrix screening is useful for broadly sampling chemical space, but if a macromolecule fails to crystallize under the precise conditions comprising a pre-formulated screen then these conditions are generally no longer pursued despite the possibility that one or more of these conditions could be optimized to yield crystals. In contrast, free-interface diffusion more thoroughly probes the crystallographic phase diagram for a given set of chemical conditions; however, its unique experimental platform is costly and makes it difficult to isolate crystals for subsequent diffraction experiments.

Several strategies have been proposed to take advantage of the data generated by automated imaging equipment in order to maximize the efficiency of crystallographic screening. For example, software programs such as *TeXRank* convert images of crystallization droplets into textons as a means of detecting the formation of ordered structures such as crystals, decreasing the time spent analyzing crystallization droplets (Leung & Malik, 2001 ▸; Ng *et al.*, 2014 ▸). Additionally, second-harmonic generation imagers have been employed to detect the formation of microcrystals that might be mistaken for, or occluded by, protein precipitation (Wampler *et al.*, 2008 ▸). Despite the abundance of information that can be gleaned from observation through automated imaging, these approaches have not yet been able to successfully convert visual data into practical information that will guide researchers towards discovering fruitful crystallization conditions (Collins *et al.*, 2017 ▸).

Here, we design and implement a highly automated method based on a series of simple calculations, guided by the crystallographic phase diagram, to iteratively optimize the precipitant concentration of each crystallization condition in a sparse-matrix screen (Haas & Drenth, 1999 ▸). After preparing an initial crystallization experiment using a 96-well sitting-drop format, a plate is incubated and automatically imaged. Five days later, each drop is manually inspected and assigned a user-generated qualitative score, which is then fed into an optimization algorithm designed to reformulate the precip­itant concentrations to achieve supersaturation. When coupled with automated liquid handling, this approach can be used to generate an optimized high-throughput crystallization screen in which the precipitant concentrations of all 96 crystallization conditions have been tuned to maximize the likelihood of crystal nucleation and growth. Because this crystallization screen will gradually become more specifically tailored towards a protein of interest after several rounds of iteration, we refer to this method as iterative screen optimization (ISO). As a proof of principle, we performed ISO to obtain crystals of a panel of six diverse protein targets and demonstrate that successive rounds of ISO identify new crystallization conditions for each protein that were not present in the original sparse-matrix screen.

## 2. Materials and methods   

### 2.1. Initial screen design   

We devised an initial crystallization screen that was confined to a set of 16 stock reagents, including water (Table 1 ▸), in an attempt to maximize the amount of chemical space that could be explored while minimizing the number of required stock reagents. We selected three polyethylene glycol (PEG) compounds ranging in molecular weight from 400 to 8000 Da, one volatile organic compound, several salts, and buffers spanning the pH range 4.6–8.5. Finally, we formulated three mixtures of chemically compatible crystallization additives, similar to the strategy successfully employed by the Morpheus screen (Molecular Dimensions; Gorrec, 2009 ▸). However, to account for the possibility that these additives may occasionally be deleterious to crystallization, we only included these cocktails as components in 35 of the 96 mother liquors that make up our final screen. Using these stock reagents, we designed 96 unique crystallization conditions to create a custom screen (Supplementary Table S1).

The design of the 96 chemical conditions that were included in the original screen was primarily restricted by the 16-ingredient limit of our automated liquid handler (Formulator 16). With this limitation in mind, we looked at successful crystallization conditions from the PDB included in the TOP96 screen (Anatrace), as well as some of the common conditions from other commercial screens, including Crystal Screen (Hampton Research) and Wizard Precipitant Synergy (Rigaku) (Jancarik & Kim, 1991 ▸; Fazio *et al.*, 2014 ▸; Majeed *et al.*, 2003 ▸). The final screen design was based on human analysis and interpretation of the most successful conditions from these sources. We then tried to develop a 96-condition screen that maximized the number of these successful conditions while remaining within the 16-stock ingredient limit. Because all 96 crystallization conditions were derived from the same set of 16 stock reagents, we refer to this novel crystallization screen as ‘Sweet16’.

### 2.2. Protein purification and preparation   

Plasmids encoding the prefusion-stabilized respiratory syncytial virus F protein (DS-Cav1; McLellan *et al.*, 2013 ▸) or the antigen-binding fragment (Fab) of the antibody motavizu­mab (Wu *et al.*, 2007 ▸) were transiently transfected into FreeStyle 293-F cells (ThermoFisher). These secreted proteins were purified from filtered cell supernatants. DS-Cav1 containing a C-terminal 8×His tag and Strep-tag II was expressed in the presence of 5 µ*M* kifunensin and was purified using Strep-Tactin resin (IBA Lifesciences). After protein elution, the affinity tags and glycans were removed by digestion with thrombin and endoglycosidase H for 2 h at room temperature. Motavizumab Fab was purified using CaptureSelect IgG-CH1 affinity matrix (Life Technologies) as per the manufacturer’s instructions. DS-Cav1 and motavizumab Fab were further purified by size-exclusion chromatography (SEC) using a Superdex 200 column (GE Healthcare) with the running buffers indicated in Table 2 ▸.

A pET-16b vector encoding an mCherry variant with an N-terminal 10×His tag was generously provided by Dr Gevorg Grigoryan (Dartmouth College). *Escherichia coli* Rosetta BL21(DE3) cells were incubated overnight in LB with ampicillin while shaking at 37°C. The bacteria were pelleted and resuspended in lysis buffer consisting of 100 m*M* imidazole pH 8.0, 20 m*M* Tris pH 8.0, 300 m*M* NaCl, 1 U universal nuclease per millilitre (Pierce) and one tablet of EDTA-free protease inhibitor per 250 ml (Roche). The cells were lysed using an M-110L microfluidizer (Microfluidics) and the lysate was centrifuged at 20 000*g* for 15 min. The protein was purified from the clarified lysate using Ni–NTA resin and was then further purified by SEC using a Superdex 75 column (GE Healthcare) in buffer consisting of 2 m*M* Tris pH 8.0, 100 m*M* NaCl, 0.02% NaN_3_. The affinity tags were removed by digestion with factor Xa for 6 h at room temperature in a buffer containing 2 m*M* CaCl_2_. The final product was separated from the cleaved tags and factor Xa by SEC using a Superdex 75 column in buffer consisting of 2 m*M* Tris pH 8.0, 100 m*M* NaCl, 0.02% NaN_3_.

Lyophilized bovine catalase, concanavalin A (Con A) and lysozyme were purchased from Sigma–Aldrich and resuspended in their respective crystallization buffers (Table 2 ▸) based on previously reported conditions (Fita & Rossmann, 1985 ▸; Hardman & Ainsworth, 1972 ▸; Alderton & Fevold, 1946 ▸).

To avoid batch-to-batch variations among our samples, all proteins were either purified from a single protein preparation or resuspended from a single commercially obtained sample. All proteins were then separated into individual aliquots and stored at −80°C. Frozen aliquots were thawed immediately prior to the preparation of a new crystallization plate.

### 2.3. Crystallization trials   

Crystallization experiments were set up using an NT8 nanovolume liquid handler (Formulatrix) and MRC 2-Well crystallization plates (Hampton Research). The initial crystallization experiment prepared using the pre-formulated Sweet16 screen is defined as ‘Plate 1’, with subsequent rounds of iteration defined as ‘Plate 2’, ‘Plate 3’ and ‘Plate 4’. In each plate, Drop 1 consisted of 100 nl protein solution and 100 nl reservoir solution and Drop 2 consisted of 100 nl protein solution and 50 nl reservoir solution. Plates were incubated at 18°C and automatically imaged in a ROCK IMAGER 1000 (Formulatrix) according to a user-defined schedule. The day on which a new crystallization experiment was dispensed is referred to as ‘Day 0’, and each crystallization plate was imaged periodically over the course of 21 days.

Under optimal circumstances, the protein concentration used for the initial Sweet16 crystallization experiment (Plate 1) yields roughly equal numbers of clear and precipitated drops. If the first plate is entirely clear or precipitated, then the best practice is to adjust the protein concentration and try again, rather than iteratively optimizing.

### 2.4. Drop scoring and screen optimization   

**Figure 1:**
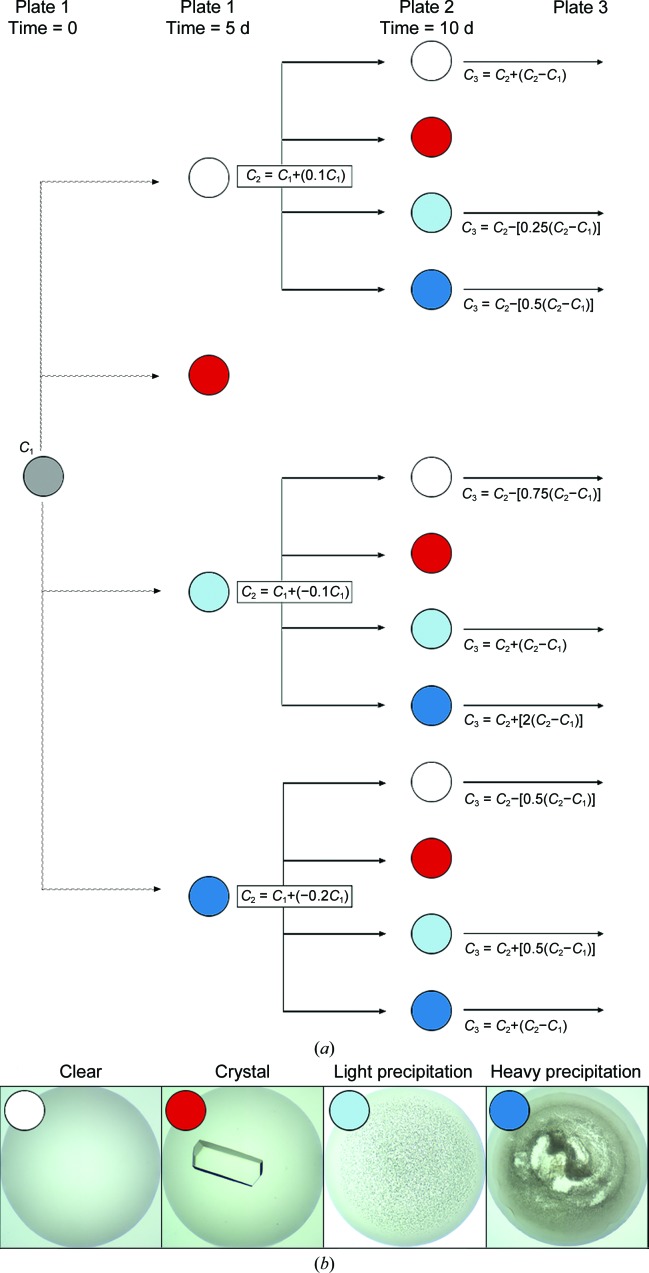
Decision tree representing the iterative screen optimization algorithm. (*a*) Drop 1 of each crystallization condition in a crystal screen is evaluated after five days and assigned a user-generated score of either ‘Clear’ (white), ‘Crystal’ (red), ‘Light Precipitation’ (light blue) or ‘Heavy Precipitation’ (dark blue). This score is then used to calculate an optimized precipitant concentration for each drop of the subsequent experiment (*C*_2_) based upon the initial precipitant concentration (*C*_1_). Once all 96 crystallization conditions have been reformulated to generate Plate 2, the optimized drops can be assigned a score after an additional five days. Further optimized precipitant concentrations (*C*_*x*_) take into account the scores of both of the two prior precipitant concentrations (*C*_*x*−1_ and *C*_*x*−2_). This process can be repeated multiple times until conditions that foster nucleation and crystal growth are found. The equations used to recalculate precipitant concentrations are listed beneath each arrow. Drops at time = 0 d have not been assigned a score and are represented by gray circles. Once conditions that result in crystal nucleation and growth have been obtained, subsequent optimization of that condition is halted. (*b*) An example from each of the possible scoring categories is shown. The circle at the top left of each image represents the score that was assigned to that drop.

Five days after the crystallization plates were dispensed, each drop was imaged and visually inspected via the *ROCK MAKER* software suite (Formulatrix). Images of Drop 1 for all 96 crystallization conditions were assigned a user-generated score of ‘Clear’, ‘Crystal’, ‘Light Precipitation’ or ‘Heavy Precipitation’, which were then input as variables into the iterative screen optimization algorithm (Fig. 1 ▸ *a*). Drops that failed to yield either crystals or precipitation were assigned a score of ‘Clear’. This scoring scheme also treated phase separation as equivalent to a ‘Clear’ drop, with the rationale that increasing the precipitant concentration is more likely to drive the protein towards nucleation and crystallization. Drops in which crystals formed were assigned the score ‘Crystal’, regardless of the morphology or quality of these crystals. This classification also included drops that yielded crystals within a background of precipitated protein, as these crystals could still be isolated for subsequent diffraction experiments or further optimized using other methods. Drops that were scored as either ‘Light Precipitation’ or ‘Heavy Precipitation’ are those in which crystals failed to form and protein aggregates precipitated out of solution. Although the distinction between ‘light’ and ‘heavy’ precipitation was somewhat subjective, this generalized scoring rubric (Fig. 1 ▸ *b*) was intended to ensure reproducibility between different users. The optimized screen was then automatically dispensed into a new crystallization plate using a FORMULATOR 16 (Formulatrix) liquid-handling device and the same set of 16 stock reagents that were used to generate the initial Sweet16 screen. A new crystallization experiment was then set up using the optimized screen, and scoring was once again performed after a five-day incubation period. Once a condition that resulted in crystal formation was reached, subsequent optimization steps were halted for that crystallization condition. Three rounds of optimization were performed for a total of four plates per protein, including the initial, unoptimized Sweet16 screen.

### 2.5. Iterative screen optimization algorithm   

The ISO algorithm uses the scores assigned during a crystallization experiment as inputs to optimize the precipitant concentrations of each condition within the crystallization screen for the subsequent experiment. When a score of ‘Clear’ is assigned to a given drop, the ISO algorithm increases the concentration of the precipitant(s) in that condition by 10% (for example, if a ‘Clear’ drop in Plate 1 has 10% PEG 400 as the precipitant, the same condition in Plate 2 would contain 11% PEG 400). Alternatively, when a score of ‘Light Precipitation’ or ‘Heavy Precipitation’ is assigned, the ISO algorithm decreases the concentration of precipitants within that condition in the subsequent screen by 10% or 20%, respectively. After multiple rounds of optimization (*i.e.* Plate 3 onwards), the ISO algorithm takes into account the scores from the two previous crystallization experiments. For example, if a condition within Plate 1 is assigned a score of ‘Clear’ and the optimized condition in Plate 2 is assigned a score of ‘Light Precipitation’, the ISO algorithm generates a further optimized condition for that drop in Plate 3 that contains a precipitant concentration between those of Plates 1 and 2. The full list of equations used to calculate optimized precipitant concentrations is shown in Supplementary Table S2. If a score of ‘Crystal’ is assigned to a drop, the precipitant concentration for that condition remains unchanged. To summarize, during the process of an ISO experiment, the stock solutions that make up each individual crystallization condition will remain the same and only the concentration of the precipitant(s) within that condition varies as a function of the user-generated qualitative score. It is also worth noting that the ISO algorithm is fully customizable in that the magnitude of each optimization step can be modified based on the requirements of a given user.

While a similar iterative optimization could theoretically be accomplished by modifying the pH rather than the precipitant concentration, implementing pH as an optimizable variable in this version of ISO would require both a high-pH and low-pH stock solution of each buffer, which would quickly exceed our solution limit of 16 reagents. To overcome this limitation, our initial Sweet16 screen design included several conditions that are chemically similar in nearly all aspects other than the pH of their buffers. For example, conditions A10, F5 and H1 all contain relatively high concentrations of PEG 4000 and 0.2 *M* ammonium sulfate, but the pH of their respective buffers ranges from 4.6 to 8.5.

### 2.6. Crystallization islands from iterative screen optimization   

The amounts of each of the three mechanistically distinct precipitants in conditions that yielded crystals were analyzed in an attempt to identify ‘crystallization islands’: clusters of successful crystallization conditions that share similar properties. The three mechanistically distinct categories of precipitant induce crystallization by altering the activity coefficient of water (salts), by increasing molecular crowding (PEG 4000 or 8000) or by altering the solvent dielectric (isopropanol, MPD or PEG 400) (Timasheff & Arakawa, 1988 ▸; Bhat & Timasheff, 1992 ▸; Majeed *et al.*, 2003 ▸). These successful crystallization conditions were plotted in three-dimensional precipitant space for each target protein and colored according to pH. Data were analyzed and plotted using the *Scatterplot*3*d* package from *R* (R Core Team, 2013 ▸). Graphs of precipitant space were analyzed for the range and diversity of crystallization islands.

## 3. Results   

To test the feasibility of ISO for general use, we selected six protein targets for crystallization that spanned a wide variety of molecular weights and higher order oligomeric compositions (Table 2 ▸). DS-Cav1, an ∼165 kDa trimeric viral glycoprotein, and motavizumab Fab, an ∼47 kDa heterodimeric immunoglobulin fragment, were both expressed in mammalian cells. mCherry, an ∼27 kDa monomeric fluorescent protein, was expressed in *E. coli*. Commercially available lysozyme, bovine catalase and Con A were also tested based on their inclusion as crystallization standards in previous evaluations of sparse-matrix crystallization screens (Jancarik & Kim, 1991 ▸; Gorrec, 2009 ▸).

**Figure 2:**
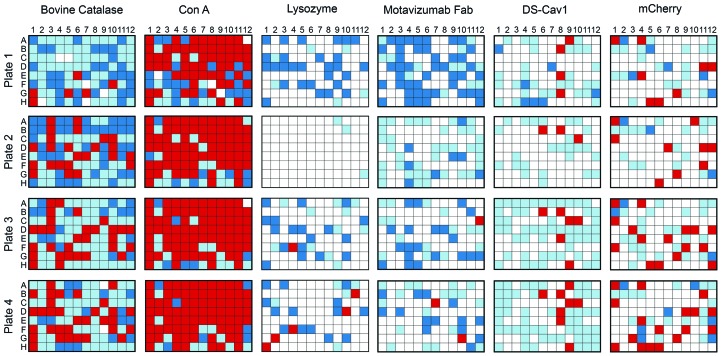
ISO results for the Sweet16 screen. The diagrams match the layout of a 96-well crystallization plate. The colors (white, clear; red, crystal; light blue, light precipitate; dark blue, heavy precipitate) indicate the score assigned to each well after a five-day incubation period.

Four of the six test proteins readily crystallized in the non-optimized Sweet16 screen (Plate 1), although the number of crystal hits varied widely between different proteins (Fig. 2 ▸). This wide variety in the number of successful crystallization conditions observed in Plate 1 illustrates the well known propensity of some proteins to crystallize with relative ease, whereas others are more resistant to crystallization (Dale *et al.*, 2003 ▸). Following a single round of ISO, most proteins showed an increase in the number of conditions that yielded crystals. Even in conditions that did not facilitate crystal growth after a single round of optimization, the effects of ISO on the level of protein precipitation could still be observed (Supplementary Fig. S1). For example, many drops that were heavily precipitated in Plate 1 were no longer scored as heavily precipitated after their precipitant concentration was decreased by 20% in the first round of optimization. The opposite effect was also observed when clear drops became either lightly or heavily precipitated after a 10% increase in precipitant concentration.

**Figure 3:**
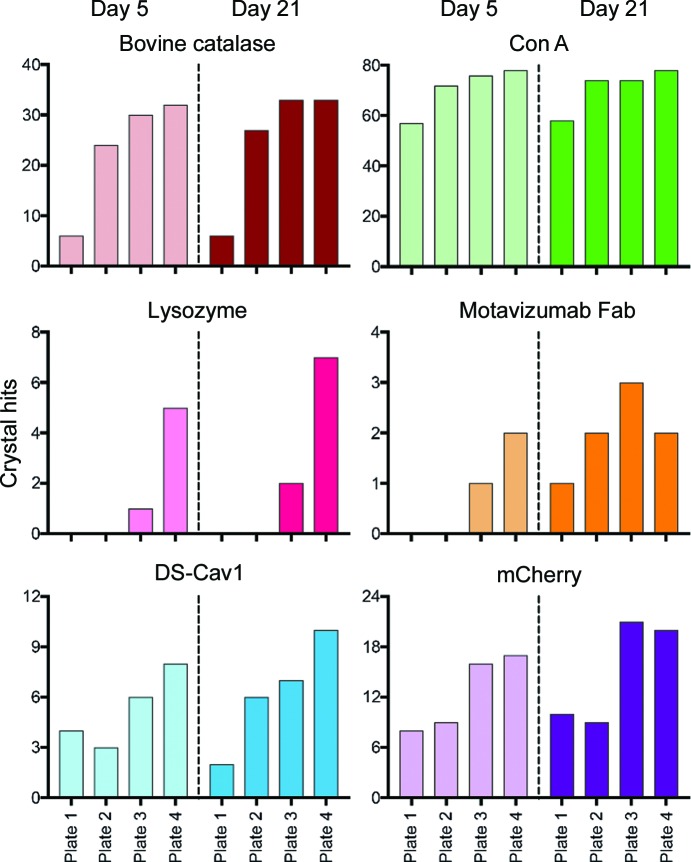
ISO results in an increase in the number of crystals obtained. The number of conditions that yielded crystals per plate for each protein is plotted on the *y* axis. Each bar along the *x* axis represents a crystallization plate, ordered 1–4 from left to right. Plate 1 refers to the initial Sweet16 screen and Plates 2–4 refer to the subsequent protein-specific optimized screens. The left half of each bar graph plots the number of conditions that yielded crystals per plate at Day 5 and the right half of each bar graph shows the number of conditions that yielded crystals per plate at Day 21.

All six of the test proteins subjected to ISO showed an increase in the number of crystallization conditions that yielded crystals after three rounds of optimization (Fig. 3 ▸). Another effect that was observed over the course of our screening was an apparent plateau in the number of conditions yielding crystals. Assuming that some chemical conditions will never be able to induce crystallization for a given protein, regardless of how thoroughly they are optimized, this plateau effect suggests that these screens are becoming maximally customized towards the protein of interest as they are subjected to multiple rounds of ISO.

As described in Section 2, user-generated scores were assigned to each drop after a five-day incubation period. A length of five days was chosen in an effort to facilitate rapid iteration, while simultaneously providing enough time for the majority of drops to equilibrate and render useful information for the ISO algorithm. Although this approach was largely effective, some drops did develop crystals after scoring on Day 5. Therefore, all plates were re-evaluated after a 21-day incubation and the number of conditions that yielded crystals was quantified (Fig. 3 ▸). Overall, the vast majority of successful crystallization conditions were identified by Day 5, although there were several instances in which crystals were present in the inspection on Day 21 that were not present on Day 5. Additionally, the trend of obtaining new, productive crystallization conditions after multiple rounds of iteration was conserved, even when evaluated at this later timepoint. This suggests that a longer incubation time prior to scoring could be helpful in identifying additional novel crystallization conditions, but five days appears to be sufficient to utilize ISO efficiently and identify the majority of fruitful crystallization conditions.

As has been well established (Forsythe *et al.*, 2002 ▸), our results indicate that the ratio of protein solution to reservoir solution is an important variable in obtaining crystals. In this set of experiments, the scores and optimization steps were based upon the first drop of each crystallization condition within the 96-well crystallization plate. This drop (Drop 1) was dispensed at a 1:1 protein:reservoir ratio, whereas the second drop (Drop 2) for each crystallization condition was dispensed at a 2:1 protein:reservoir ratio. Even though precipitant concentrations were optimized based on the scores assigned to Drop 1, we simultaneously identified a number of new, productive crystallization conditions in Drop 2 (Supplementary Fig. S2).

**Figure 4:**
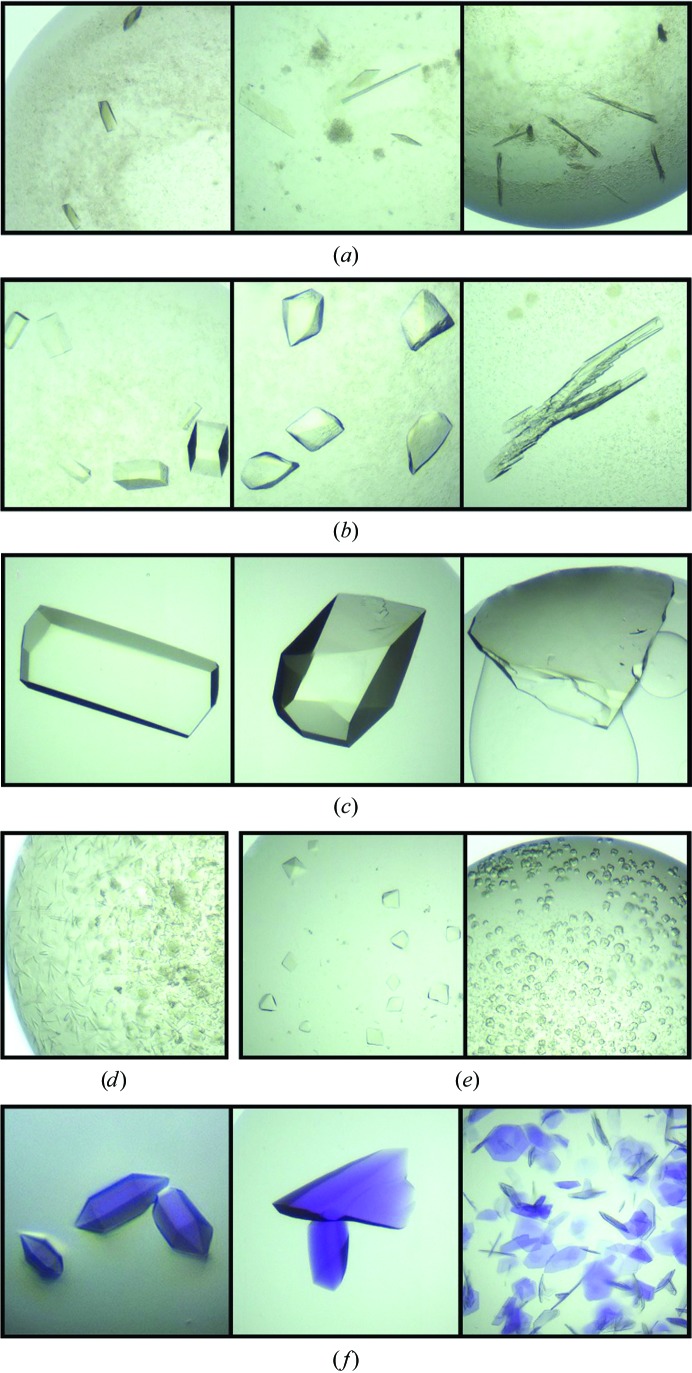
ISO yields distinct crystal morphologies. Crystals from each protein are shown in the following order: (*a*) bovine catalase, (*b*) Con A, (*c*) lysozyme, (*d*) motavizumab Fab, (*e*) DS-Cav1 and (*f*) mCherry. All images were obtained from Drop 1 after a five-day incubation period.

**Figure 5:**
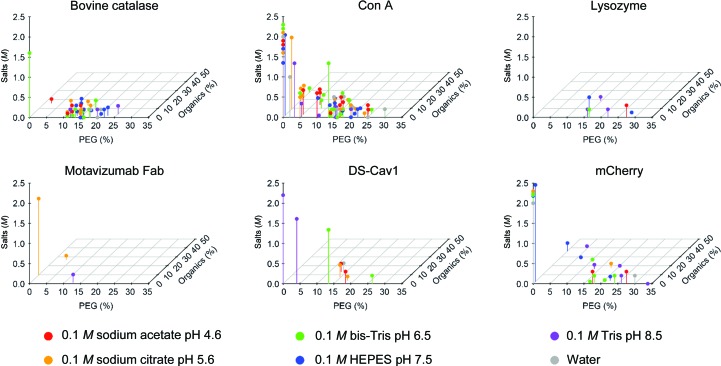
Crystallization islands for six test proteins after ISO. Each crystallization condition in Fig. 2 ▸ that yielded crystals is plotted as a single dot according to the three mechanistically distinct precipitants within that condition: salt, high-molecular-weight PEG and organic solvent. The pH of each condition is indicated by the color of the dot according to the legend (bottom). Productive crystallization conditions cluster in different ‘crystallization islands’ that range from single discrete points (*e.g.* bovine catalase at high salt, pH 6.5 in the upper left panel) to multi-condition clusters (*e.g.* mCherry with a broad range of PEG–organic conditions in the lower right panel).

Most of the proteins that were subjected to ISO formed multiple crystal morphologies (Fig. 4 ▸). This observation indicates that rather than converging on a single set of conditions which facilitate crystallization, ISO is capable of identifying multiple, distinct islands throughout chemical space that allow crystal nucleation and growth. To gain insight into the range and diversity of productive crystallization conditions, we plotted conditions that yielded crystals according to pH and the three mechanistically different precipitants: salt, high-molecular-weight PEG and organic solvent (Fig. 5 ▸). We observed distinct crystallization islands for some target proteins, such as bovine catalase, whereas the results for other proteins were more ambiguous. For example, bovine catalase demonstrated a crystallization island with one condition composed of high salt concentration (1.6 *M*) and pH 6.5 (with no PEG or organic additives), whereas another crystallization island consisted of many conditions composed of low salt concentration, moderate PEG concentrations ranging from 10 to 25% and organic additive concentrations ranging from 0 to 15%. These multiple conditions formed an extended island and covered a range of pH values. Within this larger crystallization island, crystal formation under more basic pH conditions was preferred at higher concentrations of PEG, whereas more acidic pH conditions tended to form crystals at lower PEG concentrations. A single condition at pH 4.5, 2.5% PEG and ∼15% organic additives also yielded crystals, but it was unclear whether this condition was part of the larger PEG–organic crystallization island or part of a separate, discrete island. Regarding the target protein mCherry, a similar discrete crystallization island for high salt concentrations was observed, as was an extended PEG–organic island, although a pH preference within the PEG–organic island was not readily apparent. Overall, given the possibility of obtaining a protein crystal that fails to diffract X-rays to high resolution, the multiple crystallization islands identified by ISO are a particularly attractive characteristic as they can be used to identify distinct crystallization lattices, some of which may be more suitable for diffraction experiments and structural determination.

## 4. Conclusion   

ISO is an effective method to rapidly identify multiple crystallization conditions that yield protein crystals for subsequent diffraction experiments. This screening technique requires a limited number of stock solutions, in this case only 16, which can be easily prepared using common and inexpensive reagents. Although the Sweet16 crystallization screen that we designed proved to be an effective platform for ISO, this method can be applied to any crystallographic screening strategy, regardless of the conditions that make up the initial screen. Additionally, ISO is highly customizable as almost all parameters can be varied, including the reagents making up the initial screen, the drop ratios, the incubation time before scoring and adjustments to precipitant concentrations in the ISO algorithm itself. Furthermore, the ISO platform allows users to easily experiment with and alter these parameters owing to the high degree of automation.

ISO improves upon the method of sparse-matrix screening by fine-tuning each crystallization condition in a high-throughput fashion to better suit the macromolecule that is being crystallized. Similar to free-interface diffusion, ISO is capable of thoroughly sampling chemical space, albeit over the course of multiple rounds of optimization. However, once favorable conditions have been determined, isolating crystals for diffraction experiments is far simpler in ISO because the crystals form in a standard 96-well sitting-drop plate, as opposed to forming within a capillary tube. Additionally, reproducing and optimizing productive crystallization conditions identified using ISO in a hanging-drop format should be straightforward compared with recapitulating the conditions found in free-interface diffusion. In this sense, ISO combines advantages from both sparse-matrix screening and free-interface diffusion, while maximizing the utility of a relatively small set of stock reagents.

Despite these advantages, reformulating the 96 crystallization conditions that make up a high-throughput crystallization screen during ISO without the aid of automated liquid-handling machinery would quickly become impractical. This requirement for liquid-handling machinery limits the number of stock reagents that can be used to formulate the initial, non-optimized crystallization screen. While this limitation is not prohibitive given careful screen design, it may hinder the broader sampling of potentially fruitful chemical conditions.

Future versions of ISO could incorporate automated drop-ranking programs such as *TeXRank* (Ng *et al.*, 2014 ▸) to further streamline the process and minimize the subjectivity introduced during the process of user-generated scoring. Automated optimizations could also be implemented around each crystallization island to enhance the coverage of regions of precipitant space that yield crystals. Additionally, as liquid-handling systems become more advanced, it will be possible to increase the number of stock reagents to generate more expansive initial crystallization screens. Future optimization algorithms could also include pH and drop ratios as variables rather than focusing on precipitant concentrations alone.

## Supplementary Material

Supplementary Tables and Figures.. DOI: 10.1107/S2053230X18017338/nj5280sup1.pdf

## Figures and Tables

**Table 1:** The stock solutions comprising the Sweet16 crystallization screen and their chemical compositions

	Stock	Composition
1	PEG 8000	50%(*w*/*v*) polyethylene glycol 8000
2	PEG 4000	50%(*w*/*v*) polyethylene glycol 4000
3	PEG 400	100% polyethylene glycol 400
4	MPD	100% MPD [(±)-2-methyl-2,4-pentanediol]
5	Isopropanol	100% isopropanol (isopropyl alcohol)
6	Acetates	0.5 *M* sodium acetate, 0.5 *M* calcium acetate, 0.5 *M* magnesium acetate, 0.5 *M* zinc acetate
7	Carboxylic acids	0.4 *M* sodium formate, 0.4 *M* ammonium acetate, 0.4 *M* sodium citrate, 0.4 *M* potassium sodium tartrate, 0.4 *M* sodium malonate
8	Divalent cations	1.0 *M* calcium chloride, 1.0 *M* magnesium chloride
9	Ammonium sulfate	3.5 *M* ammonium sulfate
10	Lithium sulfate	2.5 *M* lithium sulfate
11	Sodium acetate pH 4.6	1.0 *M* sodium acetate pH 4.6
12	Sodium citrate pH 5.6	1.0 *M* sodium citrate pH 5.6
13	Bis-Tris pH 6.5	1.0 *M* bis-Tris {2-[bis­(2-hydroxyethyl)amino]-2-(hydroxymethyl)propane-1,3-diol} pH 6.5
14	HEPES pH 7.5	1.0 *M* HEPES {2-[4-(2-hydroxyethyl)piperazin-1-yl]ethanesulfonic acid} pH 7.5
15	Tris pH 8.5	1.0 *M* Tris (2-amino-2-hydroxymethylpropane-1,3-diol) pH 8.5
16	Water	18.2 MΩ cm^−1^ at 25°C H_2_O

**Table 2:** The target proteins used to evaluate ISO in this study

Protein	Molecular mass (kDa)	Concentration (mg ml^−1^)	Source	Crystallization buffer
Bovine catalase	233.1 (tetramer)	10.0	Sigma–Aldrich (C9322)	2 m*M* Tris pH 8.0, 200 m*M* NaCl, 0.02% NaN_3_
Concanavalin A	25.6	11.2	Sigma–Aldrich (L7647)	2 m*M* Tris pH 8.0, 50 m*M* NaCl
Lysozyme	14.4	73.0	Sigma–Aldrich (L6876)	2 m*M* sodium acetate pH 4.6
Motavizumab Fab	47.0 (heterodimer)	9.9	McLellan laboratory	2 m*M* Tris pH 8.0, 100 m*M* NaCl, 0.02% NaN_3_
DS-Cav1	165.2 (trimer)	6.1	McLellan laboratory	2 m*M* Tris pH 8.0, 200 m*M* NaCl, 0.02% NaN_3_
mCherry	26.8	13.2	McLellan laboratory	2 m*M* Tris pH 8.0, 100 m*M* NaCl, 0.02% NaN_3_
