# Supplementary material for: Iterative screen optimization maximizes the efficiency of macromolecular crystallization
Source: Acta Crystallogr F Struct Biol Commun. 2019 Jan 24;75(Pt 2):123–31. doi: 10.1107/S2053230X18017338 (PMC6360444; doi:10.1107/S2053230X18017338)
Supplement: Supplementary file 1 [file f-75-00123-sup1.pdf]

STRUCTURAL BIOLOGY COMMUNICATIONS

Volume 75 (2019)

Supporting information for article:

Iterative screen optimization maximizes the efficiency of macromolecular crystallization

Harrison G. Jones, Daniel Wrapp, Morgan S. A. Gilman, Michael B. Battles, Nianshuang Wang, Sofia Sacerdote, Gwo-Yu Chuang, Peter D. Kwong and Jason S. McLellan

Table S1 The chemical composition for all 96 crystallization conditions in the Sweet16 screen

Acetates = equal parts sodium acetate, calcium acetate, magnesium acetate, zinc acetate. Carboxylic acids = equal parts sodium formate, ammonium acetate, sodium citrate, potassium sodium tartrate, sodium malonate. Divalent cations = equal parts calcium chloride, magnesium chloride.

Well	Precipitants	Additives	Buffers
A1	2.0 M ammonium sulfate		No buffer
A2	20% *(w/v)* PEG 4000	0.2 M acetates	No buffer
A3	20% *(w/v)* PEG 8000	0.2 M carboxylic acids	No buffer
A4	30% *(w/v)* PEG 4000	0.2 M divalent cations	No buffer
A5	25% *(w/v)* PEG 4000, 2.5% *(v/v)* MPD	0.2 M ammonium sulfate	No buffer
A6	25% *(w/v)* PEG 4000		No buffer
A7	15% *(w/v)* PEG 8000, 0.5 M lithium sulfate		No buffer
A8	2% *(w/v)* PEG 8000, 1.0 M lithium sulfate		No buffer
A9	14% *(w/v)* PEG 400, 14% *(w/v)* PEG4000	0.2 M carboxylic acids	No buffer
A10	25% *(w/v)* PEG 4000	0.2 M ammonium sulfate	0.1 M sodium acetate pH 4.6
A11	1.7 M ammonium sulfate		0.1 M sodium acetate pH 4.6
A12	30% *(v/v)* MPD	0.2 M divalent cations	0.1 M sodium acetate pH 4.6
B1	24% *(w/v)* PEG 4000, 4% *(v/v)* isopropanol		0.1 M sodium acetate pH 4.6
B2	20% *(v/v)* isopropanol	0.2 M divalent cations	0.1 M sodium acetate pH 4.6
B3	14% *(w/v)* PEG 8000		0.1 M sodium acetate pH 4.6
B4	26.8% *(v/v)* PEG 400, 3.35% *(w/v)* PEG 8000		0.1 M sodium acetate pH 4.6
B5	16.75% *(w/v)* PEG 4000, 3.35% *(v/v)* MPD	0.2 M acetates	0.1 M sodium acetate pH 4.6
B6	16.75% *(w/v)* PEG 8000	0.2 M carboxylic acids	0.1 M sodium acetate pH 4.6
B7	5% *(w/v)* PEG 4000, 3.35% *(v/v)* isopropanol, 0.5 M ammonium sulfate		0.1 M sodium acetate pH 4.6
B8	13.4% *(v/v)* PEG 400, 13.4% *(w/v)* PEG 8000	0.1 M divalent cations	0.1 M sodium acetate pH 4.6
B9	14% *(v/v)* PEG 400, 7% *(w/v)* PEG 8000	0.2 M acetates	0.1 M sodium acetate pH 4.6
B10	1.8 M lithium sulfate		0.1 M sodium acetate pH 4.6
B11	25% *(w/v)* PEG 4000	0.2 M divalent cations	0.1 M sodium acetate pH 4.6
B12	10% *(w/v)* PEG 8000, 0.5 M acetates		0.1 M sodium acetate pH 4.6
C1	2.0 M lithium sulfate		0.1 M sodium citrate pH 5.6
C2	1.0 M lithium sulfate, 0.5 M ammonium sulfate		0.1 M sodium citrate pH 5.6
C3	17% *(w/v)* PEG 8000	0.1 M carboxylic acids	0.1 M sodium citrate pH 5.6
C4	30% *(v/v)* MPD	0.2 M carboxylic acids	0.1 M sodium citrate pH 5.6
C5	20% *(v/v)* isopropanol		0.1 M sodium citrate pH 5.6
C6	6.7% *(v/v)* PEG 400, 20% *(v/v)* isopropanol	0.2 M lithium sulfate	0.1 M sodium citrate pH 5.6
C7	26.8% *(v/v)* PEG 400, 3.35% *(w/v)* PEG 4000		0.1 M sodium citrate pH 5.6
C8	20% *(v/v)* PEG 400, 2% *(v/v)* MPD	0.2 M ammonium sulfate	0.1 M sodium citrate pH 5.6
C9	3.35% *(v/v)* PEG 400, 16.75% *(w/v)* PEG 4000		0.1 M sodium citrate pH 5.6
C10	6.7% *(v/v)* PEG 400, 13.4% *(w/v)* PEG 8000	0.2 M lithium sulfate	0.1 M sodium citrate pH 5.6
C11	5% *(w/v)* PEG 4000	0.4 M sodium citrate pH 5.6	0.1 M sodium citrate pH 5.6
C12	8.04% *(v/v)* PEG 400, 1% *(v/v)* MPD, 1.675 M lithium sulfate		0.1 M sodium citrate pH 5.6

D1	13.4% *(w/v)* PEG 8000, 2% *(v/v)* MPD		0.1 M sodium citrate pH 5.6
D2	20% *(w/v)* PEG 4000, 5% *(v/v)* isopropanol		0.1 M sodium citrate pH 5.6
D3	15% *(w/v)* PEG 4000, 15% *(v/v)* MPD		0.1 M sodium citrate pH 5.6
D4	30% *(w/v)* PEG 4000		0.1 M sodium citrate pH 5.6
D5	20% *(w/v)* PEG 8000	0.2 M ammonium sulfate	0.1 M sodium citrate pH 5.6
D6	25% *(v/v)* PEG 400, 5% *(w/v)* PEG 4000		0.1 M BIS-TRIS pH 6.5
D7	30% *(w/v)* PEG 4000	0.2 M divalent cations	0.1 M BIS-TRIS pH 6.5
D8	25% *(w/v)* PEG 4000	0.2 M lithium sulfate	0.1 M BIS-TRIS pH 6.5
D9	2.3 M ammonium sulfate		0.1 M BIS-TRIS pH 6.5
D10	15% *(w/v)* PEG 8000	0.2 M divalent cations	0.1 M BIS-TRIS pH 6.5
D11	4% *(v/v)* PEG 400, 20% *(w/v)* PEG 8000		0.1 M BIS-TRIS pH 6.5
D12	2.0 M ammonium sulfate	0.2 M carboxylic acids	0.1 M BIS-TRIS pH 6.5
E1	30% *(v/v)* isopropanol	0.2 M carboxylic acids	0.1 M BIS-TRIS pH 6.5
E2	30% *(v/v)* MPD	0.2 M acetates	0.1 M BIS-TRIS pH 6.5
E3	18% *(w/v)* PEG 8000	0.2 M acetates	0.1 M BIS-TRIS pH 6.5
E4	10% *(w/v)* PEG 8000, 27% *(v/v)* isopropanol		0.1 M BIS-TRIS pH 6.5
E5	21% *(v/v)* PEG 400, 7% *(v/v)* isopropanol	0.1 M divalent cations	0.1 M BIS-TRIS pH 6.5
E6	16.75% *(w/v)* PEG 4000, 10.05% *(v/v)* MPD	0.2 M lithium sulfate	0.1 M BIS-TRIS pH 6.5
E7	13.4% *(w/v)* PEG 8000, 2.01% *(v/v)* MPD		0.1 M BIS-TRIS pH 6.5
E8	13.4% *(w/v)* PEG 4000, 1.34 M lithium sulfate		0.1 M BIS-TRIS pH 6.5
E9	25% *(w/v)* PEG 4000, 4% *(v/v)* isopropanol	0.1 M divalent cations	0.1 M BIS-TRIS pH 6.5
E10	22% *(w/v)* PEG 8000	0.2 M carboxylic acids	0.1 M BIS-TRIS pH 6.5
E11	10% *(v/v)* PEG 400, 8.5% *(w/v)* PEG 4000	0.2 M acetates	0.1 M BIS-TRIS pH 6.5
E12	20% *(w/v)* PEG 8000		0.1 M BIS-TRIS pH 6.5
F1	20% *(w/v)* PEG 8000	0.2 M divalent cations	0.1 M HEPES pH 7.5
F2	1.5 M lithium sulfate		0.1 M HEPES pH 7.5
F3	27.5% *(w/v)* PEG 4000	0.2 M lithium sulfate	0.1 M HEPES pH 7.5
F4	5% *(v/v)* PEG 400, 25% *(w/v)* PEG 4000		0.1 M HEPES pH 7.5
F5	25% *(w/v)* PEG 4000, 2.5% *(v/v)* MPD	0.2 M ammonium sulfate	0.1 M HEPES pH 7.5
F6	1.6 M ammonium sulfate	0.1 M carboxylic acids	0.1 M HEPES pH 7.5
F7	30% *(v/v)* MPD	0.2 M carboxylic acids	0.1 M HEPES pH 7.5
F8	30% *(v/v)* isopropanol	0.2 M divalent cations	0.1 M HEPES pH 7.5
F9	2% *(v/v)* PEG 400, 2.0 M ammonium sulfate		0.1 M HEPES pH 7.5
F10	10.05% *(w/v)* PEG 4000, 13.4% *(v/v)* isopropanol		0.1 M HEPES pH 7.5
F11	26.8% *(v/v)* PEG 400, 5.36% *(w/v)* PEG 8000		0.1 M HEPES pH 7.5
F12	16.75% *(w/v)* PEG 4000, 2.68% *(v/v)* isopropanol	0.1 M divalent cations	0.1 M HEPES pH 7.5
G1	12% *(w/v)* PEG 8000, 12% *(v/v)* MPD	0.2 M lithium sulfate	0.1 M HEPES pH 7.5
G2	15% *(w/v)* PEG 8000, 0.5 M carboxylic acids		0.1 M HEPES pH 7.5
G3	28% *(v/v)* PEG 400	0.2 M divalent cations	0.1 M HEPES pH 7.5
G4	25% *(w/v)* PEG 4000		0.1 M HEPES pH 7.5
G5	13.4% *(w/v)* PEG 8000, 6.7% *(v/v)* isopropanol	0.2 M ammonium sulfate	0.1 M HEPES pH 7.5
G6	20% *(w/v)* PEG 4000, 10% *(v/v)* isopropanol		0.1 M HEPES pH 7.5
G7	25% *(w/v)* PEG 4000, 4% *(v/v)* isopropanol	0.2 M carboxylic acids	0.1 M TRIS pH 8.5
G8	2.2 M ammonium sulfate		0.1 M TRIS pH 8.5
G9	3% *(v/v)* PEG 400, 30% *(v/v)* MPD		0.1 M TRIS pH 8.5

G10	25% *(w/v)* PEG 8000, 2.5% *(v/v)* MPD	0.2 M lithium sulfate	0.1 M TRIS pH 8.5
G11	20% *(w/v)* PEG 8000	0.2 M divalent cations	0.1 M TRIS pH 8.5
G12	5% *(v/v)* PEG 400, 25% *(w/v)* PEG 8000	0.1 M acetates	0.1 M TRIS pH 8.5
H1	20% *(w/v)* PEG 4000	0.2 M ammonium sulfate	0.1 M TRIS pH 8.5
H2	30% *(v/v)* PEG 400	0.2 M carboxylic acids	0.1 M TRIS pH 8.5
H3	30% *(v/v)* isopropanol	0.2 M carboxylic acids	0.1 M TRIS pH 8.5
H4	26% *(w/v)* PEG 8000		0.1 M TRIS pH 8.5
H5	20% *(w/v)* PEG 4000, 20% *(v/v)* isopropanol		0.1 M TRIS pH 8.5
H6	16.75% *(v/v)* PEG 400, 13.4% *(w/v)* PEG 4000	0.1 M divalent cations	0.1 M TRIS pH 8.5
H7	20.1% *(v/v)* PEG 400, 5.36% *(v/v)* MPD		0.1 M TRIS pH 8.5
H8	6% *(w/v)* PEG 8000, 0.335 M carboxylic acids		0.1 M TRIS pH 8.5
H9	3.35% *(w/v)* PEG 4000, 1.34 M lithium sulfate		0.1 M TRIS pH 8.5
H10	9% *(w/v)* PEG 4000, 9% *(v/v)* MPD		0.1 M TRIS pH 8.5
H11	10% *(w/v)* PEG 4000, 2% *(v/v)* MPD		0.1 M TRIS pH 8.5
H12	35% *(v/v)* PEG 400, 3.5% *(w/v)* PEG 8000		0.1 M TRIS pH 8.5

Table S2 Precipitant optimization equations.

The first three equations determine the optimized precipitant concentration of a condition in Plate 2 (C_2) based on the assigned score and the initial precipitant concentration (C_1). Any further optimization steps are determined by the lower equations. These equations calculate an optimized precipitant concentration (C_x) based on the two previously assigned scores and the two previous precipitant concentrations (C_{x-1} and C_{x-2}).

Assigned Score(s)	Equation
Clear	$C_2=C_1+(0.1C_1)$
Light Precipitation	$C_2=C_1+(-0.1C_1)$
Heavy Precipitation	$C_2=C_1+(-0.2C_1)$
Clear → Clear	$C_x=C_{x-1}+(C_{x-1}-C_{x-2})$
Clear → Light Precipitation	$C_x=C_{x-1}-(0.25(C_{x-1}-C_{x-2}))$
Clear → Heavy Precipitation	$C_x=C_{x-1}-(0.5(C_{x-1}-C_{x-2}))$
Light Precipitation → Clear	$C_x=C_{x-1}-(0.75(C_{x-1}-C_{x-2}))$
Light Precipitation → Light Precipitation	$C_x=C_{x-1}+(C_{x-1}-C_{x-2})$
Light Precipitation → Heavy Precipitation	$C_x=C_{x-1}+(2(C_{x-1}-C_{x-2}))$
Heavy Precipitation → Clear	$C_x=C_{x-1}-(0.5(C_{x-1}-C_{x-2}))$
Heavy Precipitation → Light Precipitation	$C_x=C_{x-1}+(0.5(C_{x-1}-C_{x-2}))$
Heavy Precipitation → Heavy Precipitation	$C_x=C_{x-1}+(C_{x-1}-C_{x-2})$

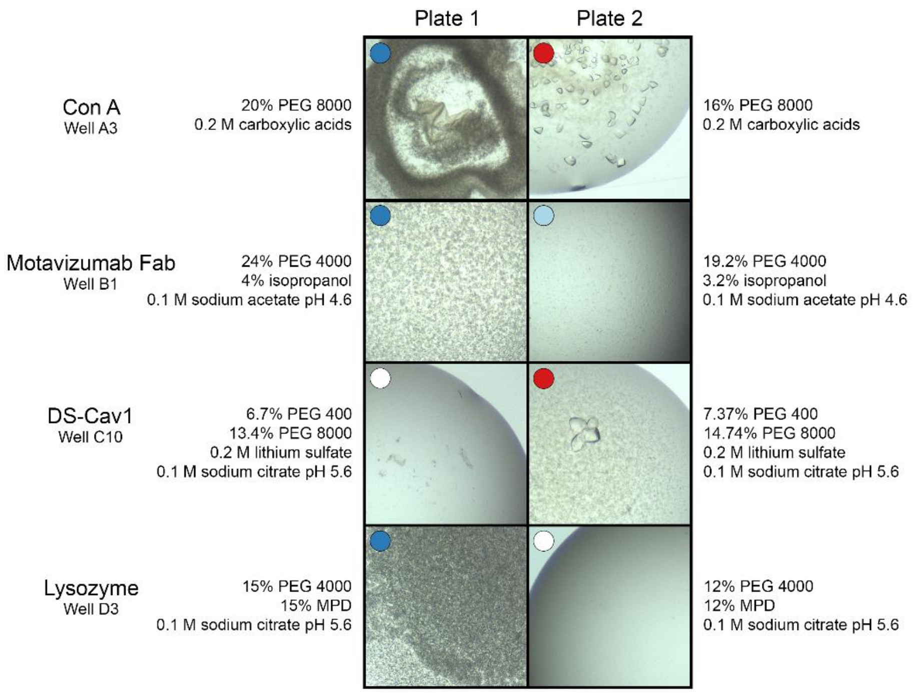

Figure S1 Effects of ISO after a single round of optimization. Drops from the initial Sweet16 screen (Plate 1) are shown (*left*) and the same drops from Plate 2 are shown after a single round of scoring and optimization (*right*). The protein, well number and composition of the crystallization condition are listed alongside each image. The colored circle in the top left of each image represents the score that was assigned to that drop. The coloring scheme for these scores matches that of **Figure 1**. All images were taken of Drop 1 after a five-day incubation period.

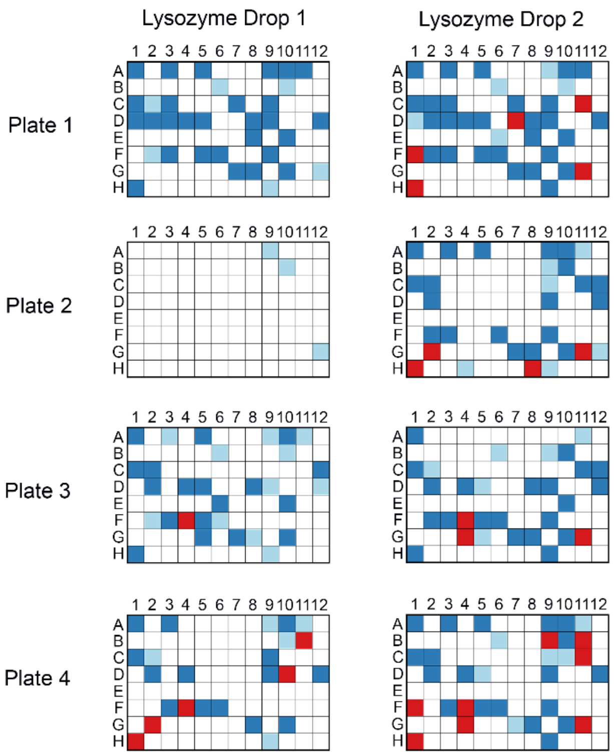

Figure S2 Optimizing precipitant concentrations based on the results of Drop 1 can improve the results of Drop 2. Plate diagrams are shown for lysozyme Drop 1 (*left*) and Drop 2 (*right*). Drop 1 was dispensed at a ratio of 100 nL:100 nL (protein:reservoir) and Drop 2 was dispensed at a ratio of 100 nL:50 nL (protein:reservoir). Precipitant optimization calculations were performed based on the scores assigned to Drop 1 after five days. The coloring scheme used for scoring matches that of **Figure 1**.
